# Effectiveness and safety of Belimumab combined with standard therapy in severe active lupus nephritis requiring kidney replacement therapy: A case report and literature review

**DOI:** 10.3389/fimmu.2022.977377

**Published:** 2022-09-12

**Authors:** Chengning Zhang, Ming Zeng, Yifei Ge, Kang Liu, Changying Xing, Huijuan Mao

**Affiliations:** Department of Nephrology, The First Affiliated Hospital of Nanjing Medical University (Jiangsu Province Hospital), Nanjing, China

**Keywords:** severe active lupus nephritis, belimumab, acute kidney injury, kidney replacement therapy, case report

## Abstract

Lupus Nephritis (LN) is the most common manifestation of severe organ damage for systemic lupus erythematosus (SLE) patients. Severe active LN could result in acute kidney injury (AKI), which could even require Kidney Replacement Therapy (KRT). Therefore, there needs to be a more proactive and safe induction therapy to quickly and effectively control renal immune inflammation, maintain kidney function or reverse kidney damage. While multiple clinical studies have proven the efficacy and safety of Belimumab in treating SLE and LN, these studies have not included cases of severe LN requiring KRT. We observed the effectiveness and safety of Belimumab in treating four severe active LN patients undergoing KRT. With Belimumab administered at a dosage of 10mg/kg, all four patients were able to discontinue KRT with no adverse events (AEs) to date ultimately. These cases provided an excellent basis for the application of Belimumab combined with standard therapy to LN patients with a medium to severe kidney injury.

## Introduction

Systemic lupus erythematosus (SLE) is a chronic connective tissue disease characterized by immune inflammation which could affect all body systems. Persistent disease activity or frequent flares could result in progressive organ damage and poorer quality of life (1). Lupus nephritis (LN), the most common form of severe organ damage for SLE patients, is the primary cause of death from the disease. 40% to 60% of adult SLE patients have presented with LN over the disease course (2). Severe LN flares could result in acute kidney injury (AKI) and could even require Kidney Replacement Therapy (KRT). Therefore, there needs to be a more proactive and safe induction therapy to quickly and effectively control renal immune inflammation, maintain kidney function or reverse kidney damage.

The abnormal activation of B lymphocytes, which results in the overproduction of autoantibodies against various cytoplasmic and nuclear antigens, is a key component in the pathogenesis of SLE. As a member of the tumor necrosis factor family, the B lymphocyte stimulator (BLyS)/B-cell activating factor (BAFF) is mainly expressed by myeloid cells and its main function is to stimulate the proliferation, differentiation and antibody secretion of B lymphocytes. BLyS overexpression in active SLE/LN patients results in the survival of autoreactive B cells, which were supposed to have undergone apoptosis (3, 4). These autoreactive B cells then circulate in the body to produce autoantibodies, leading to tissue inflammation and damage (5). Therefore, a potential key therapeutic target for SLE is to prevent the binding of BLyS to B cell surface receptors to inhibit the survival and differentiation of B cells.

Belimumab is a human IgG-1λ monoclonal antibody that specifically binds to soluble BLyS to inhibit the differentiation of B cells and reduce the number of activated B cells and plasma cells. In the BLISS-52, BLISS-76, BLISS-SC and BLISS-Northeast Asia clinical studies on Belimumab therapy for adult SLE patients, it was found that Belimumab improve SLE Responder Index-4 (SRI-4) response rate and lower SLE activity while also reducing flares and dose of glucocorticoids (6–9). With good safety features, Belimumab is approved for patients with active autoantibody-positive SLE who are 5 years or older. In the 2020 BLISS-LN study, more patients with active LN who received Belimumab combined with standard therapy achieved Primary Efficacy Renal Response (PERR) and Complete Renal Response (CRR) than those who received standard therapy alone (10). The findings are proof of Belimumab’s safety and efficacy in treating LN. However, the exclusion criteria for the BLISS-LN study included patients who had undergone dialysis within one year or an estimated glomerular filtration rate (eGFR) of less than 30 mL/min/1.73m^2^. The eGFR at baseline of the two groups enrolled in this study was 101.0 ± 42.7 and 100.0 ± 37.7 mL/min/1.73m^2^, respectively, and the urine protein/creatinine (uPCR) was 3.5 ± 3.6 and 3.2± 2.7 g/g, which could not cover the full clinical picture of LN. In this article, we report on the treatment of four patients with severe LN requiring KRT with Belimumab to observe the effectiveness and safety of Belimumab in this group of patients.

## Case descriptions

There were 94 patients diagnosed with LN by renal pathology in the Department of Nephrology in The First Affiliated Hospital of Nanjing Medical University from December 2020 to May 2022, 12 of whom required KRT. Three of the 12 patients had a pathological chronicity index score of 12 and two had severe infections, so a total of five patients did not use Belimumab. The remaining 7 patients received standard therapy in combination with Belimumab, 3 of whom discontinued treatment or lost follow-up due to high treatment costs and the novel coronavirus outbreak, and 4 patients on continuous therapy are summarized here. Besides Standard Therapy, Belimumab was administered at a dosage of 10mg/kg at day 1, day 15, day 29 and subsequently once every 28 days. During the follow-up duration between 7 to 13 months (as of May 2022), all four patients were able to discontinue KRT.

### Case 1

An 18-year-old Chinese young man attended hospital with 3 months of facial erythema and 1 month of edema. Facial erythema was primarily distributed on both sides of the nose bridge, accompanied by hair loss, severe swelling of the lower limbs, and shortness of breath after activity. The laboratory tests showed blood routine: White blood cells (WBC) 2.80*10^9/L, hemoglobin (HGB) 75g/l, platelets (PLT) 85*10^9/L; urine protein 4+, urine occult blood 3+; urinary red blood cell (RBC) morphology: RBC count 4 million/ml, dysmorphic RBC 75%; C3 0.172g/l (reference range, 0.790-1.520g/L), C4 0.0350g/l (reference range, 0.160-0.380g/L), antinuclear antibodies (ANA) >1:3200, anti-double stranded DNA (anti-dsDNA) titer 750.8IU/ml. Cardiac ultrasound showed small amount of pericardial effusion, LVEF was 34%. The pathological type of LN was Class III and V. Systemic Lupus Erythematosus Disease Activity Index 2000 (SLEDAI-2K) score was 22. Renal function progressed to urea 40.82 mmol/l and creatinine 228.3 umol/l. At the same time the patient had chest tightness, 24-hour urine output decreased to 500-700 ml. Bioelectrical impedance analysis (BIA) showed the overhydration (OH) value was +11.8L. Continuous Veno-Venous Hemofiltration (CVVH) was performed for ultrafiltration for dehydration. Methylprednisolone (MP) pulse therapy was given and continued with prednisone tablets. Tacrolimus was chosen considering the potential reproductive toxicity of CTX for this young man. Prednisone was slowly tapered to a maintained dosage of 5 mg/day. A total of 12 doses Belimumab therapy were administered.

### Case 2

A 63-year-old Chinese woman presented to the hospital with joint pain accompanied by bilateral lower limbs swelling for six months, nausea and vomiting for 1 week. The laboratory tests showed blood routine: WBC 4.58*10^9/L, HGB 73g/l, PLT 240*10^9/L; urine protein 3+, urine occult blood 1+; urinary RBC morphology: RBC count 84,000/ml, dysmorphic RBC 70%; C3 0.284g/l, C4 0.0173g/l, ANA >1:1000, anti-dsDNA titer 697.9IU/ml. The pathological type of LN was Class IV and V. SLEDAI-2K score was 20. Cardiac ultrasound showed a moderate amount of pericardial effusion. Renal function progressed to urea 42.39mmol/l and creatinine 365.7 umol/l. The 24-hour urine output decreased to 400ml, and BIA showed the OH value was +13.0L. Continuous renal replacement therapy (CRRT) was initiated. MP pulse therapy was given and continued with prednisone tablets combined with cyclophosphamide (CTX). Prednisone was slowly tapered to a maintained dosage of 10 mg/day in combination with tacrolimus. A total of 8 doses Belimumab therapy were administered.

### Case 3

A 46-year-old Chinese woman was admitted to the hospital with two months of edema of the eyelids and lower limbs. The laboratory tests showed blood routine: WBC 5.25*10^9/L, HGB 70g/l, PLT 37*10^9/L; urine protein 3+, urine occult blood 1+; urinary RBC morphology: RBC count 68,000/ml, dysmorphic RBC 70%; C3 0.251g/l, C4 0.106g/l, ANA >1:1000, anti-dsDNA titer 17.9IU/ml. Pathological type of LN was Class IV and V. SLEDAI-2K score was 24. During the course of the disease, the patient gradually developed apathy, unresponsiveness and drowsiness. Renal function progressed to urea 30.8 mmol/l and creatinine 155.5 umol/l. The 24-hour urine output decreased to 500ml, and BIA showed the OH value was +6.0L. At the same time, serum sodium rose to 165umol/l, so CRRT was initiated. After MP pulse therapy, the methylprednisolone dosage was reduced to 60mg/day. Meanwhile, gamma globulin infusions were performed to improve immunity together with rituximab (100mg/time x 2 times). After treatment, the patient’s consciousness and renal function improved. Half a month later, the patient developed a severe pulmonary infection and acute respiratory distress syndrome (ARDS), with pneumocystis carinii infection, recurring decreased urine output and acute kidney injury. Co-trimoxazole was administered, and the prednisone was slowly tapered to a maintained dosage of 10 mg/day. A total of 7 doses Belimumab therapy were administered after infection control.

### Case 4

A 48-year-old Chinese man was admitted to the hospital with two months of bilateral lower limbs edema and one month of dyspnea. The laboratory tests showed blood routine WBC 3.01*10^9/L, HGB 72g/l, PLT 63*10^9/L; urine protein 4+, urine occult blood 3+; urinary RBC morphology: RBC count 320,000/ml, dysmorphic RBC 75%; C3 0.203g/l, C4 0.0364g/l, ANA 1:1000, anti-dsDNA titer 331.6IU/ml. The pathological type of LN was Class IV. SLEDAI-2K score was 19. Renal function progressed to urea 28.94 mmol/l and creatinine 455.3 umol/l. The 24-hour urine output decreased to 600-700ml and BIA showed the OH value was +9.0L. CRRT was initiated. The patient also received plasma exchanges and MP pulse therapy. It was continued to administer prednisone plus CTX, and the prednisone was slowly tapered to 10 mg/day. A total of 4 doses Belimumab therapy were administered.

Characteristics of patients before and after Belimumab treatment were shown in **Table 1**.

**Table 1:** Characteristics of patients before and after Belimumab treatment.

		Case 1	Case 2	Case 3	Case 4
Gender		Male	Female	Female	Male
Age (years)		18	63	46	48
Follow-up (months)		12	10	13	7
Pathology		Class III+V	Class IV+V	Class IV+V	Class IV
Histologic score	AI	17	23	16	19
	CI	4	6	4	2
SLEDA I-2K Score	Before	22	20	24	19
	After	2	6	10	10
24h urine protein(mg)	Before	7893	9611	12264	6917
	After	85	1302	334	2374
Albumin level (g/L)	Before	21.7	27.4	23	31.1
	After	39.8	37.8	31.8	33
eGFR (ml/min/1.73m^2^)	Before	34.98	10.76	34.1	12.21
	After	138.59	30.46	85.05	22.88
Anti-dsDNA titer (IU/mL)	Before	750.8	697.9	17.9	331.6
	After	2.0	2.0	2.0	2.0
C3(g/L)	Before	0.172	0.284	0.251	0.203
	After	0.631	0.675	0.687	0.757
C4(g/L)	Before	0.0350	0.0173	0.106	0.0364
	After	0.162	0.141	0.219	0.202
Prednisone dose(mg)	Before	60	30	35	40
	After	5	10	10	10
Standard Therapy		Prednisone and tacrolimus	Prednisone, CTX and tacrolimus	Prednisone	Prednisone and CTX
Combination therapy other than Belimumab		–	–	Low dose RTX	Plasma exchange
Duration of KRT (days)		7	10	150	210

AI, Activity Index; CI, Chronicity Index; CTX, Cyclophosphamide; RTX, Rituximab; KRT, Kidney Replacement Therapy.

## Discussion

The kidney is the most commonly involved organ among SLE patients. In China, nearly half of the SLE patient population have concurrent LN, with an incidence rate higher than that of Caucasians (11). Over the past few decades, patient and kidney survival rates for LN have improved significantly with the advent of new treatment strategies. However, despite these advances, up to 30% of LN patients progress to end-stage renal disease (ESRD). Therefore, a more proactive and safe induction therapy is needed to control renal immune inflammation and maintain kidney function or reverse kidney damage.

MA Dooley et al. performed a pooled post-hoc analysis on the 1,684 active LN patients of the BLISS 52/76 studies in order to evaluate the effect of Belimumab on the renal parameters in patients with baseline renal involvement. The findings showed that over 52 weeks, although the between-group differences in most renal outcomes were not significant, rates of renal flare, renal remission, renal organ disease improvement, proteinuria reduction, and serologic activity favored Belimumab. However, there were certain limitations in this analysis, including the small number of patients and the post-hoc nature of the pooled analysis (12). To date, BLISS-LN is currently the largest LN study in the world with 448 patients recruited. The results showed that at Week 104, compared with standard therapy alone, the Belimumab group in the induction course performed significantly better in terms of renal remission rate, risk of renal-related events or death, reduction in proteinuria, the proportion of renal relapse, effective maintenance of eGFR stability, reduction in disease activity, and administered hormone dosages below 7.5 mg/day. Meanwhile, the AE rates of the Belimumab group were similar to the placebo trial group (10). Meta-analysis showed that the probability of the Belimumab trial group achieving a CRR was 1.71 times that of the control group. There were no significant differences in the incidence of treatment-related AEs and serious AEs between the two groups (13). In terms of the efficacy and safety of Belimumab in treating LN, the U.S. Food & Drug Administration (FDA) approved Belimumab to treat adults with active LN who are receiving standard therapy in December 2020 (14).

All four patients in this paper were severe active LN and had received KRT at the onset of the disease. Severe renal damage portends inferior patient survival, and ESRD is associated with a 26–63-fold increase in the standardized mortality ratio. The objective of treatment for severe active LN is to preserve nephrons by reversing the acute inflammatory process and achieving a state of disease quiescence (15). Currently, the effectiveness and safety of Belimumab in severe LN requiring KRT has not been reported in the literature. These patients have massive proteinuria and severe renal impairment, it is uncertain whether Belimumab requires dosage adjustments when used. It has been reported in the literature that massive proteinuria in LN results in the loss of monoclonal antibodies including immunoglobulins in urine. To take Belimumab for instance, the Belimumab exposure would be 50% lower in patients with uPCR of 5 g/g compared to patients without proteinuria. Further analysis found that patients with a baseline uPCR of <1.24g/g responded significantly faster to Belimumab, while patients with higher baseline proteinuria had similar responses as the patients in the control group (14). This is consistent with the BLISS-LN post-hoc analysis findings (16). While proteinuria levels evidently affect the efficacy of Belimumab, it does not mean that Belimumab has no activity in LN patients with high proteinuria. The post-hoc analysis of the high proteinuria group (uPCR≥3g/g) showed that the Belimumab trial group had a lower estimated risk of renal-related event or death than those in the placebo group at Week 104. As BLISS-LN only evaluated the 10 mg/kg IV Q4W dosing regimen, there is currently insufficient data to support the notion that increasing the Belimumab dosage in low exposure dose patients (such as those with high proteinuria) would increase the drug response rate or shorten the response duration among LN patients.

Although the patients enrolled in the BLISS-LN study had active LN, they only had a mildly decrease in renal function. Therefore, there have been no conclusive findings on how to use Belimumab to treat patients with severely reduced renal function due to active LN especially those receiving KRT (17). It has been found that all the B cell subtypes of ESRD patients decrease except for transitional B cells, and the decreased expression of BAFF receptors on B cells leads to a decrease in total B cells. In turn, this may generate high levels of BAFF through a positive feedback mechanism (18). A case of refractory SLE patient entering maintenance hemodialysis for LN treated with Belimumab to control extra-renal lupus activity was reported in Japan. Belimumab treatment was chosen considering the side effects of increasing hormones and previous use of intravenous CTX and mycophenolate mofetil. The first and second dose after a 2-week interval was 10 mg/kg. Complement levels increased after the first treatment and anti-dsDNA titer decreased after 1 month. However, the patient developed Pneumocystis carinii pneumonia and congestive heart failure after 3 weeks, and the dose of Belimumab was reduced from 10 mg/kg to 4 mg/kg after the patient’s condition improved due to concerns about the risk of reinfection. After 7 doses of Belimumab, the SLEDAI score decreased to 4, and the only reported AE was pneumonia (19). An Italian patient with SLE on peritoneal dialysis for ESRD was treated with Belimumab. After a few months, the patient’s arthralgia was resolved, immunological parameters improved, and hormone dose was reduced. No adverse reactions took place during the treatment course (20). The cases above shows that despite having entered maintenance dialysis, Belimumab is still an excellent treatment option for extrarenal lupus activity in SLE patients. However, special care should be paid to preventing infection during use, as Belimumab and uremia synergistically inhibit B cell lineage through different pathways (19). While all four of our cases had massive proteinuria and hypoalbuminemia at the onset, they also had concomitant acute kidney injury and decreased urine output. Theoretically, these conditions might mutually offset the state of Belimumab exposure, and the general hemodialysis pattern had no additional clearance of Belimumab. Therefore, we still used the conventional dose of 10 mg/kg and achieved pretty good treatment outcomes, whereby severe renal impairment was significantly improved without any serious side effects upon undergoing Belimumab plus Standard Therapy. These cases showed that Belimumab can be used in patients with moderate to severe renal impairment under conditions of close monitoring for infection prevention. However, further pharmacokinetic and clinical studies are needed to ascertain if dose adjustments are required for the use of antibody-based biologics in this patient population.

The Case 3 patient in this paper presented with concomitant lupus encephalopathy. At the start, she was treated with hormone therapy plus low dose Rituximab at a 100 mg x 2 times regimen (administered at a half-month interval). Half a month after receiving the second dose of Rituximab, the patient developed a fever, cough with sputum, and decreased pulse oxygen, which was clearly diagnosed as “pneumocystis carinii pneumonia”. After undergoing anti-infective treatment, the patient was put on a revised hormone therapy regimen plus Belimumab for seven months and had yet to show any adverse reactions. Furthermore, a case of SLE with cytomegalovirus pneumonia, bone marrow suppression, and relapse of SLE with further increase in proteinuria and creatinine during standard treatment was reported. The patient was treated with Belimumab (10 mg/kg/month intravenously) considering the high risk of the previous infection, and after 12 months the indicators improved significantly and the SLEDAI score decreased. The treatment was switched to subcutaneous injection (200 mg/week) and complete renal remission was achieved after 11 months, with no side effects or infections reported during the 30-month follow-up period, and the final maintenance dose of hormone was 5 mg/day (21). These two cases suggest that Belimumab is a relatively safer and more effective option for LN patients with a high risk of infection.

There are currently no guideline recommendations for the course of Belimumab for LN. IN Bruce et al. analyzed the patients in the BLISS 52/76 study with long-term follow-up, with the primary endpoint being the change in the Systemic Lupus International Collaborating Clinics Damage Index (SDI) from baseline at years 5-6. The findings showed that SLE patients who received long-term Belimumab plus Standard Therapy had a lower incidence of organ damage and no unexpected AE (22). A 13-year follow-up study showed that Belimumab was well tolerated with no new safety concerns in the treatment of SLE, and patients who responded to Belimumab on initial treatment maintained good tolerance and disease control over time (23). Evidently, the long-term usage of this drug to control SLE is safe and efficacious. However, the safety and efficacy of Belimumab for long-term treatment of LN has not been reported in the literature and further clinical studies are needed to verify this.

The treatment outcomes of the four patients in this paper show that even in patients with severe active LN requiring KRT, the administration of Belimumab 10 mg/kg can still bring better therapeutic effect with better safety. Aggressive control of LN activity can maximize the protection of renal function. The safety and efficacy of Belimumab in LN patients with moderate to severe renal impairment and whether the dose and duration of treatment need to be adjusted need to be further explored in clinical studies with large number of patients.

## Data availability statement

The original contributions presented in the study are included in the article. Further inquiries can be directed to the corresponding author.

## Ethics statement

Ethical review and approval was not required for the study on human participants in accordance with the local legislation and institutional requirements. The patients/participants provided their written informed consent to participate in this study. Written informed consent was obtained from the individual(s) for the publication of any potentially identifiable images or data included in this article.

## Author contributions

CZ collected clinical data, summarized the cases, reviewed the literature, and drafted the manuscript. MZ, YG and KL provided the cases. CX and HM edited the manuscript. All authors contributed to the article and approved the submitted version.

## Funding

This work was supported by National Natural Science Foundation of China (Grant Number: 81970639, 82151320) and Priority Academic Program Development of Jiangsu Higher Education Institutions (CN).

## Acknowledgments

The authors wish to thank the patients in this study.

## Conflict of interest

The authors declare that the research was conducted in the absence of any commercial or financial relationships that could be construed as a potential conflict of interest.

## Publisher’s note

All claims expressed in this article are solely those of the authors and do not necessarily represent those of their affiliated organizations, or those of the publisher, the editors and the reviewers. Any product that may be evaluated in this article, or claim that may be made by its manufacturer, is not guaranteed or endorsed by the publisher.
